# Adenovirus type 5 SARS-CoV-2 vaccines delivered orally or intranasally reduced disease severity and transmission in a hamster model

**DOI:** 10.1126/scitranslmed.abn6868

**Published:** 2022-05-05

**Authors:** Stephanie N. Langel, Susan Johnson, Clarissa I. Martinez, Sarah N. Tedjakusuma, Nadine Peinovich, Emery G. Dora, Philip J. Kuehl, Hammad Irshad, Edward G. Barrett, Adam Werts, Sean N Tucker

**Affiliations:** ^1^ Duke Center for Human Systems Immunology and Department of Surgery, Durham, NC 27710, USA; ^2^ Vaxart, South San Francisco, CA 94080, USA; ^3^ Lovelace Biomedical Research Institute, Albuquerque, NM 87108, USA

## Abstract

Transmission-blocking strategies that slow the spread of severe acute respiratory syndrome coronavirus 2 (SARS-CoV-2) and protect against coronavirus disease 2019 (COVID-19) are needed. We have developed an orally-delivered Adenovirus type (Ad) 5-vectored SARS-CoV-2 vaccine candidate that expresses the spike protein. Here we demonstrated that hamsters vaccinated by the oral or intranasal route had robust and cross-reactive antibody responses. We then induced a post-vaccination infection by inoculating vaccinated hamsters with SARS-CoV-2. Oral- or intranasal-vaccinated hamsters had decreased viral RNA and infectious virus in the nose and lungs and experienced less lung pathology compared to mock-vaccinated hamsters after SARS-CoV-2 challenge. Naïve hamsters exposed in a unidirectional air flow chamber to mucosally-vaccinated, SARS-CoV-2-infected hamsters also had lower nasal swab viral RNA and exhibited fewer clinical symptoms than control animals, suggesting that the mucosal-route reduced viral transmission. The same platform encoding the SARS-CoV-2 spike and nucleocapsid proteins elicited mucosal cross-reactive SARS-CoV-2-specific IgA responses in a phase 1 clinical trial (NCT04563702). Our data demonstrate that mucosal immunization is a viable strategy to decrease SARS-CoV-2 disease and airborne transmission.

## Teaser

Oral and intranasal adenovirus 5-vectored SARS-CoV-2 vaccines reduced disease and decreased airborne transmission in a hamster model.

## INTRODUCTION

The intramuscular severe acute respiratory syndrome coronavirus 2 (SARS-CoV-2) vaccines currently approved for clinical use are capable of protecting vaccinees from symptomatic infection, hospitalization, and death from coronavirus disease 2019 (COVID-19) (*1*–*3*). However, they do not completely prevent infection. Indeed, mRNA vaccinated individuals infected with the B.1.617.2 (Delta) and B.1.1.529 (Omicron) variant can shed viral RNA and infectious virus and potentially spread SARS-CoV-2 to others (*4*–*6*). The Omicron variant appears more capable of avoiding vaccine-induced immunity than the Delta variant (*7*), and caused a substantial winter surge in infections in the United States, creating an acute shortage of healthcare workers (*8*). Considering most of the world is under immunized, including all children under 5 and majority of children 5 to 12 years of age, the possibility that a vaccinated individual with a post-vaccination infection can spread SARS-CoV-2 to unimmunized family or community members poses a public health risk. There would be a substantial benefit to develop vaccines that protect against disease and reduce SARS-CoV-2 transmission from vaccinated to unvaccinated individuals.

Since the mucosal surface of the upper respiratory tract (URT) is the initial site of SARS-CoV-2 replication and primary site of infection (*9*), interventions that induce robust mucosal immune responses may have the greatest impact on reduction of SARS-CoV-2 transmission. We have created a replication-defective, shelf-stable oral adenoviral type 5 (Ad5) vector vaccine candidate expressing the spike (S) protein from SARS-CoV-2 (r-Ad-S) that is designed to induce both systemic and mucosal immunity. Over 500 human participants have been administered these oral Ad5 vaccines, which have been well-tolerated and able to generate robust humoral and cellular immune responses to the expressed antigens (*10*–*13*). Importantly, immune activation through the intestine may represent an important organ for oral immunization, as antibody secreting plasmablasts and plasma cells can traffic from the gut to the nose, trachea, and lung (*14*, *15*). Prior work in a human influenza challenge study with the same platform has shown an ability to limit viral RNA shedding of influenza virus (*11*), highlighting the utility of this vaccination strategy for respiratory viruses.

To study the potential impact of oral vaccination on transmission to naïve individuals, we used a hamster infection model and unidirectional air flow chambers. We vaccinated index hamsters with oral r-Ad-S, using intranasal (IN) r-Ad-S as a control for mucosal stimulation, intramuscular spike protein (IM S) as a protein control, and oral phosphate-buffered saline (PBS) as a mock control. We then infected animals intranasally with a high titer of SARS-CoV-2 in order to replicate a post-vaccination infection. One-day post viral challenge, index hamsters were placed upstream of vaccine-naïve hamsters in a chamber that allowed airborne movement but not direct contact or fomite transmission. Here we report the clinical and virological responses of both the vaccinated (SARS-CoV-2 infected) and naïve (SARS-CoV-2 exposed) hamsters. Additionally, we present mucosal antibody data from participants in a phase 1 clinical trial using the same platform encoding the SARS-CoV-2 spike and nucleocapsid proteins. These data demonstrate that oral r-Ad-S immunization resulted in reduced disease and decreased SARS-CoV-2 transmission in a hamster model and can generate coronavirus cross-reactive, spike protein-specific IgA in the nose and mouths of humans.

## RESULTS

### Oral and IN r-Ad-S vaccination induced robust systemic and mucosal antibody responses.

**Fig. 1.**
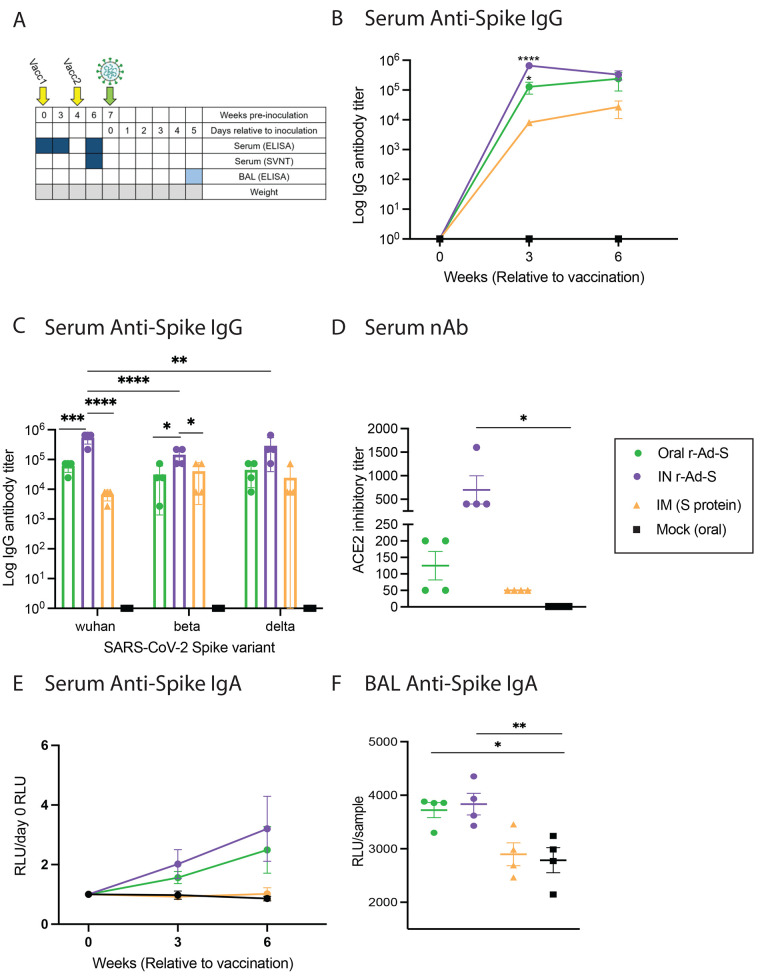
Oral and intranasal r-Ad-S vaccination induced robust IgG and IgA antibodies in golden hamsters. Index hamsters were immunized with oral r-Ad-S, intranasal (IN) r-Ad-S, intramuscular (IM) spike protein (S) or mock (oral) (*n* = 4 per group) and inoculated with SARS-CoV-2 seven weeks later. (**A**) Experimental design schematic. Figure created with BioRender.com (**B**) Serum anti-spike protein IgG antibody endpoint titers were measured at weeks 0, 3 and 6 post immunization by enzyme-linked immunosorbent assay (ELISA). (**C**) Serum anti-spike protein IgG antibody endpoint titers were measured at week 6 post immunization against the spike protein of the Wuhan, Beta, and Delta SARS-CoV-2 variants by ELISA. (**D**) Surrogate neutralizing antibodies (antibodies capable of blocking binding of SARS-CoV-2 spike protein to ACE2) titers were measured in serum at week 6. (**E**) Serum anti-spike protein IgA antibody titers were measured (MSD arbitrary units (AU)/sample) at weeks 0, 3, and 6 post immunization using the MSD platform and normalized to day 0 AU values. (**F**) anti-spike protein IgA antibodies were measured in BAL fluid using the MSD platform. Data were analyzed by a one-way ANOVA and Dunnett’s multiple comparisons. Comparisons were made between vaccinated groups and mock (oral) controls for (B, D, E and F). Error bars represent the standard error of the mean (SEM). *P<0.05, **P<0.01, ***P<0.001, ****P<0.0001.

Index hamsters were immunized at weeks 0 and 4 with oral r-Ad-S, IN r-Ad-S (mucosal positive control), IM spike protein or mock (oral PBS) prior to SARS-CoV-2 challenge at week 7 (Fig. 1A). To determine immunogenicity of these vaccines, serum was collected at weeks 0, 3 and 6 post immunization. Bronchoalveolar lavage (BAL) fluid was collected upon necropsy (day 5 post-inoculation) (Fig. 1A). Oral and IN r-Ad-S vaccinated groups had significantly higher spike protein-specific IgG antibody titers in serum at week 3 compared to mock-dosed hamsters (p=0.0165, Oral; p<0.0001, IN); this was not true in IM spike protein-vaccinated hamsters (Fig. 1B). Serum spike protein-specific IgG at 6 weeks post immunization was also cross-reactive to the Beta and Delta variants in all groups (Fig. 1C). Using a surrogate virus neutralizing test (sVNT), we found that serum from IN r-Ad-S hamsters had greater ability to block binding of SARS-CoV-2 spike protein to angiotensin converting enzyme 2 (ACE-2) after the booster vaccination (week 6) compared to serum from mock-vaccinated hamsters (Fig. 1D). Serum anti-spike protein IgA antibodies increased post-oral and IN r-Ad-S vaccination but not in IM spike protein or mock vaccinated groups (Fig. 1E). As expected from our oral immunization platform, oral r-Ad-S vaccinated hamsters demonstrated similar spike protein-specific IgA concentrations in BAL fluid compared to the IN r-Ad-S positive control group, suggesting similar stimulation of mucosal immunity (Fig. 1F). There were no differences in serum or BAL IgA responses between IM spike protein and mock-vaccinated hamsters, suggesting that IgA was not induced by systemic immunization with the IM spike protein. These data demonstrate that oral r-Ad-S and IN r-Ad-S vaccinated hamsters generated robust systemic and mucosal humoral immunity.

### Oral and IN r-Ad-S vaccination accelerated SARS-CoV-2 viral RNA clearance and protected against disease in hamsters.

**Fig. 2.**
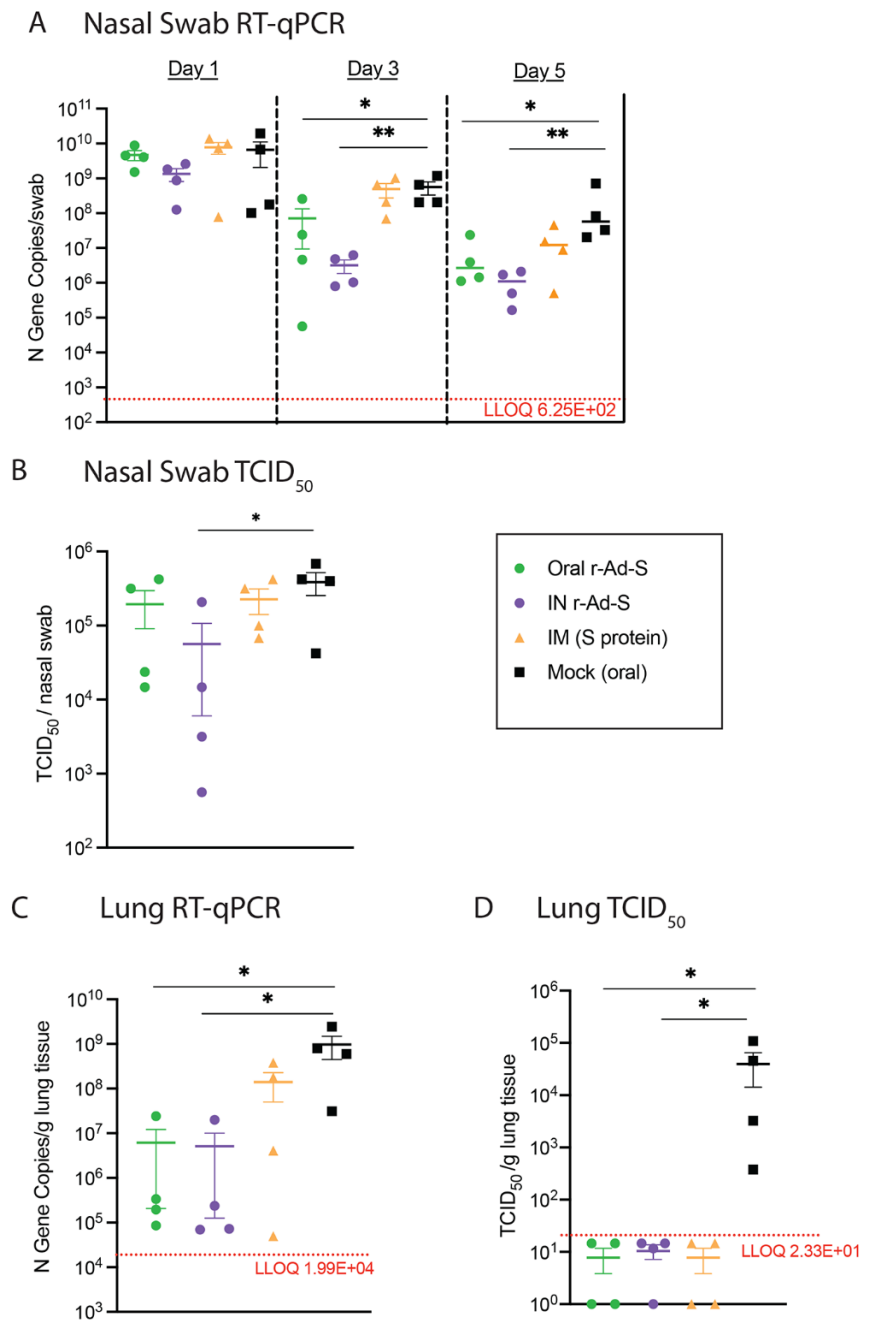
Oral and intranasal r-Ad-S vaccination decreased SARS-CoV-2 RNA and infectious virus in nasal swabs and lungs. (**A**) Nasal swabs were collected on days 1, 3, and 5 post-inoculation in index hamsters (*n* = 4 per group) and viral genomic RNA loads in these samples were determined by quantitative reverse transcription PCR (qRT-PCR) of the N gene. (**B**) Nasal swabs were collected on day 1 post inoculation and infectious virus titers were determined by TCID_50_. (**C**) Lung tissue was collected at necropsy (day 5) and RNA was isolated for SARS-CoV-2 detection by qRT-PCR of the N gene and (**D**) infectious viral titers by TCID_50_. The dotted line represents Lower Limit of Quantification (LLOQ), with data below the limit of detection plotted at ½ LLOQ. Data were analyzed by a one-way ANOVA and Dunnett’s multiple comparisons (A to C) or Kruskal-Wallis with Dunn’s multiple comparisons (D). Comparisons were made between vaccinated groups and mock (oral) controls. Error bars represent the SEM. *P<0.05, **P<0.01.

In index animals, SARS-CoV-2 RNA from nasal swabs at 3 and 5 days post-inoculation was lower in oral and IN r-Ad-S-vaccinated animals compared to mock-vaccinated animals; this was not true for IM-vaccinated animals (Fig. 2A). Nasal swabs of index animals immunized with IN r-Ad-S had significantly lower median tissue culture infectious dose (TCID_50_) values compared to mock animals on day 1 (p=0.0377, Fig. 2B), but all groups still had detectable infectious virus. Oral and IN r-Ad-S-vaccinated index animals had significantly lower viral RNA loads in the lungs at terminal collection (day 5 post inoculation) when compared to mock-vaccinated animals (p=0.0198, Oral; p=0.0135, IN), which was not true for IM spike protein-vaccinated animals (Fig. 2C). All vaccinees had significantly reduced lung infectious viral loads compared to mock (p<0.0001), with all appearing below the limit of detection at day 5 (Fig. 2D). These data demonstrated that oral and IN r-Ad-S vaccination decreased SARS-CoV-2 post-infection and accelerated viral clearance.

**Fig. 3.**
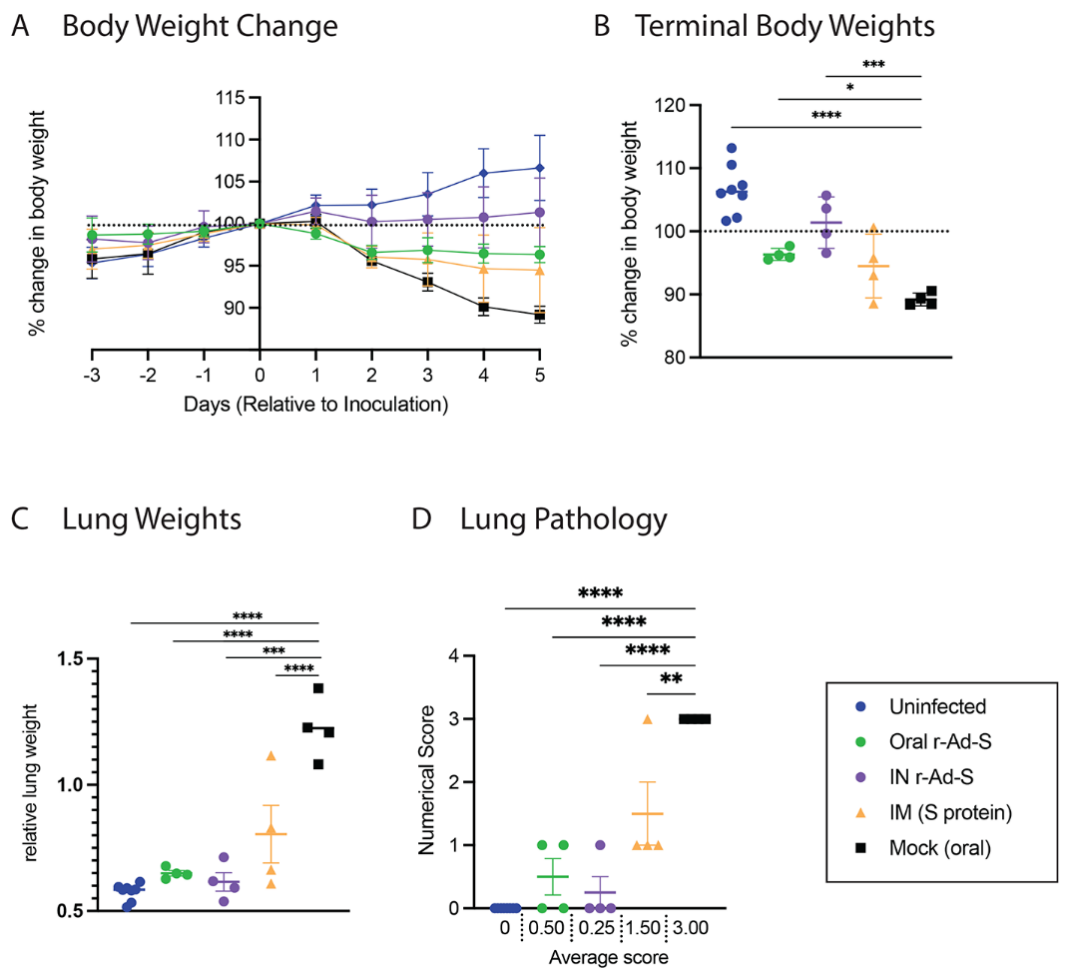
Oral and intranasal r-Ad-S vaccination reduced disease indicators, including body weight loss, lung weight, and lung pathology scores in index hamsters. (**A**) Daily weight change and (**B**) terminal body weights were determined by the percent of day 0 weight (relative to SARS-CoV-2 inoculation) for index hamsters (*n* = 4 per group). (**C**) relative terminal lung weights and (**D**) lung pathology scores were determined. Severity grade for red discoloration of the lung was based on a 0 to 4 scale indicating percent of whole lung affected: none (no grade), minimal (1), mild (2), moderate (3), marked (4) correlating to 0, 1 to 25, 26 to 50, 51 to 75, or 76 to 100% affected, respectively. Data were analyzed by a one-way ANOVA and Dunnett’s multiple comparisons. Comparisons were made between vaccinated groups and mock (oral) controls. Error bars represent the SEM. *P<0.05, **P<0.01, ***P<0.001, ****P<0.0001.

During vaccination, but prior to SARS-CoV-2 inoculation, all animals were gaining weight at a similar rate (fig. S1). After SARS-CoV-2 inoculation in index hamsters, we observed significantly greater weight loss in unvaccinated hamsters (AUC = 767 ± 2.5) compared to uninfected hamsters (AUC = 809.6 ± 4.0) (p<0.0001, Fig. 3A), a characteristic of disease in this model. Oral and IN r-Ad-S vaccinated animals lost less weight by the termination of the study compared to mock-vaccinated animals (Fig. 3B). To quantify pulmonary inflammation, lung weights were measured, and lungs were scored for gross pathology. In index animals, lung weights (normalized to total body weight) (Fig. 3C) and average gross pathology scores (Fig. 3D) were decreased in both oral and IN r-Ad-S-vaccinated groups when compared to mock-treated hamsters.

### Oral and IN r-Ad-S vaccination limited SARS-CoV-2 transmission to unvaccinated, naïve hamsters leading to decreased clinical evidence of disease.

As a test of transmissibility, unvaccinated naïve hamsters were exposed to vaccinated, SARS-CoV-2-infected index animals at a 1:4 ratio of index to naïve, where each vaccine exposure group has 4 index animals and 16 vaccine naïve animals. Exposure was performed by putting an index animal in a chamber (index chamber) connected to a second chamber containing the four naïve animals (naïve chamber), separated by a 5 inch connecting chamber (fig. S2). Screens at either end of the connecting chamber prevented direct contact. Air was circulated in the index chamber by fan, and was pulled into the naïve chamber by vacuum. Exposure of naïve hamsters to air flow produced by index hamsters was performed for 8 hours prior to moving each hamster to an individual cage. On day 1 post infection the amount of viral RNA loads in all index animals was above 7.8x10^7^ gene copies per swab (Fig. 2A).

**Fig. 4.**
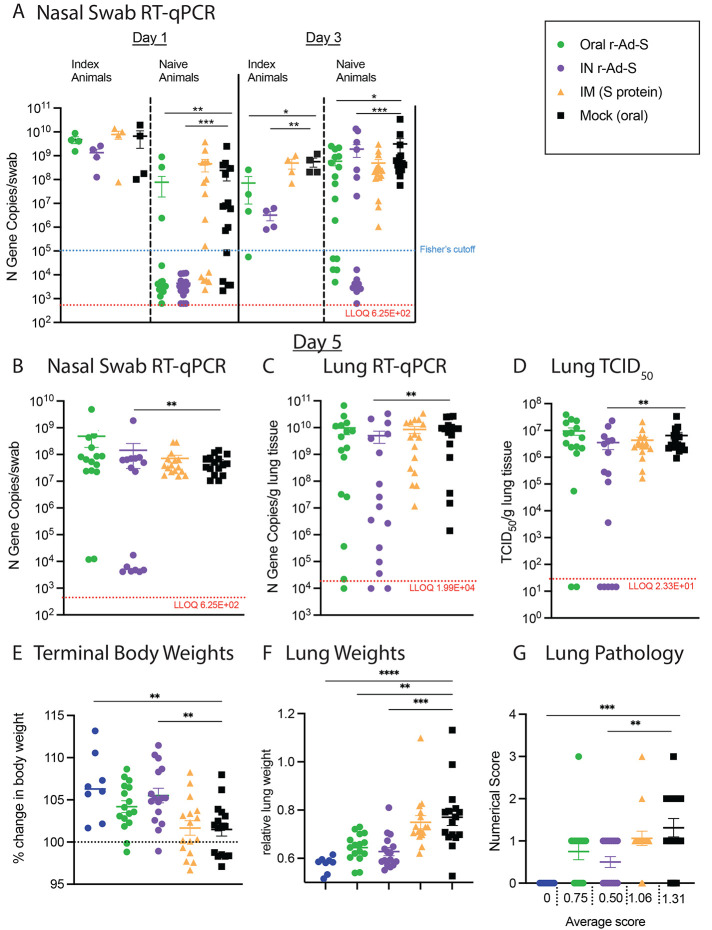
Oral and intranasal SARS-CoV-2 vaccination decreased SARS-CoV-2 aerosol transmission. (**A**) Nasal swabs were collected on days 1 and 3 post-inoculation from index animals (*n* = 4 per group) and from naïve animals (*n* = 16 per group) on days 1, 3, and (**B**) 5 after exposure to index, infected hamsters in airborne transmission chambers. Viral genomic RNA loads in these samples were determined by qRT-PCR of the N gene. (**C**) Lung tissue was collected at necropsy (day 5) and RNA was isolated for SARS-CoV-2 detection by qRT-PCR of the N gene. (**D**) Infectious viral titers in lung tissue were determined by TCID_50_. In (**A to D**) The dotted line represents the LLOQ, with data below the limit of detection plotted at ½ LLOQ. (**E**) Terminal body weights were determined by the percent weight change versus day 0 (relative to SARS-CoV-2 inoculation). (**F**) Terminal lung weights and (**G**) lung pathology scores were determined. Severity grade for red discoloration of the lung was based on a 0 to 4 scale as in Fig. 3D. In (**E to G**), data were analyzed by a one-way ANOVA and Dunnett’s multiple comparisons. Comparisons were made between vaccinated groups and mock (oral) controls. For all panels, error bars represent the SEM. *P <0.05, **P <0.01, ***P < 0.001, ****P<0.0001.

In naïve hamsters exposed to the oral and IN r-Ad-S-immunized groups, SARS-CoV-2 RNA was significantly lower on days 1 and 3 compared to hamsters exposed to mock-immunized animals (p=0.0075, Oral day 1; p=0.0001, IN day 1; p=0.0367, Oral day 3; p=0.0001, IN day 3; Fig. 4A). The number of vaccine-naïve animals with nasal swab viral RNA above a threshold of 1x10^5^ gene copies was also determined. On day 1 post exposure of the index and naïve animals, there were 3 naïve hamsters (3/16) exposed to the oral r-Ad-S index group that were above the threshold compared to eleven mock exposed hamsters (11/16) (p=0.011, Fisher’s exact test) (Fig. 4A, table S1). The naïve hamsters exposed to IN r-Ad-S index hamsters had 0 (0/16) animals above the threshold, which was not significantly different than oral vaccination, but was significantly lower than naïve animals exposed to airborne transmission from mock-vaccination animals (p=0.22, p<0.0001 by Fisher’s exact test respectively) (Fig. 4A, table S1). On day 3, in the group exposed to orally-vaccinated hamsters, 11 (11/16) had nasal swab viral RNA concentrations above the threshold compared to 16 (16/16) for the group exposed to unvaccinated index hamsters (p=0.043 by Fisher’s exact test) (Fig. 4A, table S1). The naïve animals exposed to IN-vaccinated index animals had 7/16 animals with nasal swab viral RNA above the threshold, which was not significantly different than the animals exposed to orally-vaccinated index hamsters (p=0.285 by Fisher’s exact test) (Fig. 4A, table S1). These data demonstrate decreased SARS-CoV-2 transmission from oral and IN r-Ad-S**-**vaccinated index to naïve animals. On day 5, nasal viral RNA did not differ between naïve hamsters exposed to orally-vaccinated index and control index hamsters. However, nasal swab and lung viral RNA (p=0.0062 and p=0.0077, respectively) and infectious virus (p=0.002) were lower in hamsters exposed to IN r-Ad-S index hamsters compared to those exposed to unvaccinated index hamsters (Fig. 4B to D).

Although oral and IN r-Ad-S vaccination did not completely prevent transmission, the vaccination likely reduced the effective dose reaching the naïve animals. In a prior virus titering experiment, we demonstrated animals lost more weight and had increased pulmonary inflammation when they received higher titers of SARS-CoV-2 inoculum, suggesting that the severity of disease acquired is dependent on the original infectious dose (fig. S3). Similar dose-dependent disease severity findings have been shown by others in hamsters, mice, ferrets, and nonhuman primates (*16*–*20*). Reduction in effective dose may have led to a greater proportion of naïve animals exposed to oral r-Ad-S (14/16) and IN r-Ad-S (15/16) vaccinated hamsters gaining weight by the end of the study (Fig. 4E). This is compared to naïve animals exposed to the IM (S) vaccinated (8/16) and unvaccinated (10/16) hamsters (Fig. 4E) where fewer naïve animals increased in size. Additionally, hamsters exposed to oral and IN r-Ad-S-vaccinated index hamsters had significantly lower lung weights (p= 0.0012, Oral, 0.0002, IN, Fig. 4F). Additionally, hamsters exposed to IN r-Ad-S-vaccinated index hamsters had lower pathology (p=0.0049 IN, Fig. 4G) scores compared to unvaccinated hamsters. These data demonstrate that oral and IN r-Ad-S-vaccinated animals with a post-vaccination SARS-CoV-2 infection transmit less infectious virus through the air to unvaccinated, naïve hamsters than infected but unvaccinated (or IM vaccinated) animals, and that this difference in transmission resulted in reduced evidence of severe disease.

### Oral Ad5 vaccination in humans elicits cross-reactive IgA.

**Fig. 5.**
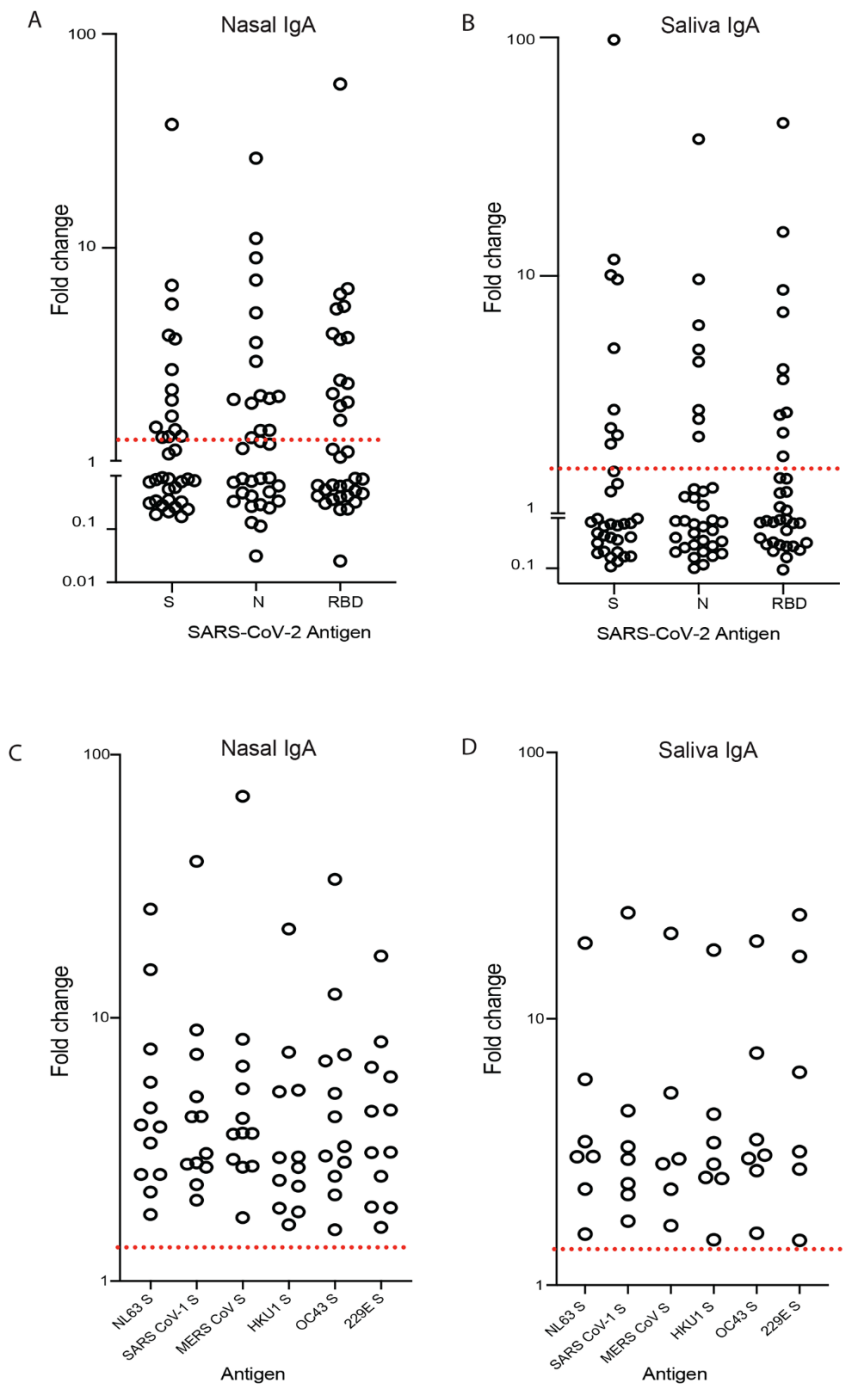
Cross reactive anti-spike protein IgA in the mucosal and salivary compartments is elicited by oral Ad5 vaccination in a subset of participants. (**A and B**) Fold change (day 29 versus day 1) in spike (S)-, nucleocapsid (N)-, or receptor binding domain (RBD)-specific IgA antibodies in nasal (A) and saliva (B) samples were measured by the MSD platform (*n* = 35). (**C and D**). The fold change in IgA specific to endemic human coronaviruses was measured in both nasal (C) and saliva (D) samples respectively, in the responders (*n* = 12 or n=7 respectively) from (A) and (B). The red dotted line represents a two-fold change, by which individuals were classified as responders.

Using the same platform in an open-label phase 1 clinical study, 35 healthy individuals received either a single low (1x10^10^ infectious units (IU); n=15) or high (5x10^10^ IU; n=15) dose of the oral tablet vaccine candidate VXA-CoV2-1 (encoding the SARS-CoV-2 spike and nucleocapsid proteins); additionally, a small cohort (n=5) received two low doses. SARS-CoV-2 specific IgA was measured in the mucosal compartment using the Mesoscale discovery (MSD) platform. 54% of vaccinees (19/35) had a 2-fold or higher increase in mucosal IgA in either saliva or nasal samples (Fig. 5A and B). More specifically, 10/35 (29%) of vaccinees had a 2-fold or higher increase in IgA antibodies in their saliva, whereas 12/35 (35%) reached the same threshold in their nasal compartment by day 29 post vaccination (Fig. 5A and B). The responses were similar to all three antigens measured: S, N, and the receptor binding domain (RBD). Furthermore, those that had a 2-fold increase in saliva or nasal virus-specific IgA also showed an increase in cross-reactive IgA, which bound to spike proteins from all 4 endemic coronaviruses, middle east respiratory syndrome coronavirus (MERS-CoV) and SARS-CoV-1 (Fig. 5C and D). We did not observe differences between the two doses.

## DISCUSSION

SARS-CoV-2 emerged in late 2019 and quickly spread around the globe, leading to hundreds of millions of cases and over 4 million deaths. Despite reports of decreased viral RNA shedding from mRNA-vaccinated individuals compared to unvaccinated individuals (*5*, *21*), recent evidence has demonstrated that vaccinated individuals can get infected with SARS-CoV-2 and shed infectious virus, which can lead to onward transmission. Improved mucosal responses that stimulate local immunity at the site of SARS-CoV-2 replication may have a much greater impact on human transmission. In this study, we show that oral and IN r-Ad-S vaccination decreased evidence of severe disease in vaccinated hamsters and reduced SARS-CoV-2 transmission to unvaccinated, naïve hamsters.

We observed robust anti-spike protein IgG responses after oral and IN r-Ad-S vaccination, as previously demonstrated in a different oral r-Ad-S hamster experiment (*22*). Further, we could detect elevated IgA in the serum and the BAL fluid of mucosally-immunized animals. The mucosally-vaccinated animals had reduced airborne transmission to naïve animals during an 8-hour airborne exposure window. Specifically, this was assessed by reduced nasal swab viral RNA loads in naïve animals one and three days post transmission exposure to oral and IN r-Ad-S-vaccinated hamsters as compared to control exposed hamsters. We hypothesize that mucosal antibodies in the URT were able to enhance SARS-CoV-2 clearance in vaccinated animals, limiting the infectiousness of transmitted aerosols. Consistent with this hypothesis, anti-spike protein IgA in the BAL fluid of the oral and IN r-Ad-S immunized animals was higher than IM protein- or mock-vaccinated animals.

Other groups have also tested SARS-CoV-2 transmission in a hamster model. For example, van Doremalen and colleagues determined that IN-vaccinated hamsters were protected from SARS-CoV-2 when placed in the same cage as unvaccinated hamsters inoculated with 1x10^4^ TCID_50_ of virus (*18*). These experiments determined whether IN-vaccinated animals were protected against transmission from unvaccinated animals. In our study, we tested whether mucosal vaccination reduces the ability of the virus replicating in vaccinated individuals from being transmitted specifically through the air to naïve animals. We used a relatively high dose of virus to induce a post-vaccine infection (1x10^5^ TCID_50_) and physically separated animals in the exposure chamber to remove the possibility of contact or fomite exposure as a means of transmission. We showed that mucosal vaccination could reduce SARS-CoV-2 transmission from vaccinated to unvaccinated animals. These data are relevant because of its implication in global public health, especially in areas in which a high percentage of the people are unvaccinated. Additionally, we demonstrated that serum IgG from all vaccinated hamsters bound to spike protein of both the Beta and Delta variants of concern. This suggests that mucosal vaccination could induce cross-protective antibodies against new variants of concerns. In support of that, we presented data that demonstrates that immunization with an oral tablet spike and nucleocapsid protein vaccine, VXA-COV2-1, generated robust anti-spike protein IgA in nasal swabs and saliva in a subset of individuals, which bound to the spike proteins of multiple coronaviruses, including the four endemic human coronaviruses (NL63, HKU1, OC43, and 229E) and other pathogenic coronaviruses (MERS-CoV, SARS-CoV-1). It is possible that mucosal vaccination against SARS-CoV-2 induces IgA antibodies at the mucosal surface that have superior cross-reactivity to coronaviruses when compared to systemic IgG antibodies. Indeed, reports have shown IgA to dominate the early neutralizing antibodies response to SARS-CoV-2 (*23*) and may be more broadly cross-reactive against various coronaviruses (*24*, *25*). Additionally, it is suggested that anti-influenza IgA antibodies generated at the mucosal surface are more broadly reactive, likely resulting from enhanced avidity from their multimeric (mostly dimeric) structure (*26*–*28*). If additional variants, such as Omicron, arise that result in an increase in post-vaccine infections, broadly-reactive IgA antibodies that contribute to reducing transmission even in vaccinated populations would help to limit circulating virus. Mucosal vaccination could be considered for implementation not only because they protect the vaccinee, but because they likely have a greater effect on the community as a whole. Reducing SARS-CoV-2 transmission to the unprotected is likely to lead to decreased hospitalization and deaths.

Our study does have limitations. First, we did not measure mucosal T cell responses, which have been shown to play a role in limiting SARS-CoV-2 infection at mucosal sites (*29*). Additionally, the SARS-CoV-2 challenge dose we used was above physiological dose likely to be picked up by an environmental exposure, as evidenced by the high viral RNA load in the nose at 1-day post infection in all index hamsters. It is likely that mucosal immunization would provide greater protection against SARS-CoV-2 transmission when lower doses of challenge virus are used. Our study was meant to be a stringent challenge to clearly identify advantages between vaccine groups. For practical reasons, such as the limitation of the number of airborne transmission chambers, lower dose virus inoculation or evaluation at later time points were not done. Additionally, future work should include challenging mucosally-vaccinated hamsters with the Delta and Omicron variants and other variants of concern. In terms of future human studies, a clinical trial with the vaccine construct used in this hamster study is predicted to improve the mucosal antibody response rate, as studies in non-human primates have shown improved antibody responses compared to the original vaccine tested in humans (*30*).

An orally-delivered, temperature-stable SARS-CoV-2 vaccine is ideal for global vaccination, where adequate storage and qualified health care providers may be in short supply. IN delivery has some of the same advantages but translating IN SARS-CoV-2 vaccination efficacy in humans has proven to be more difficult than in animals (*31*, *32*). Implementing oral vaccine campaigns around the world has been done, as evidenced by the rotavirus and poliovirus vaccination efforts (*33*, *34*). We have previously demonstrated that our SARS-CoV-2 clinical candidate vaccine, VXA-COV2-1, generated robust humoral immune responses in mice (*35*). Additionally, it was well-tolerated and immunogenic in a phase 1 clinical trial where the oral vaccine was delivered to participants as tablets (NCT04563702) and protected hamsters from SARS-CoV-2 challenge when given by oral gavage (*22*). An additional phase 2 study using the vaccine candidate tested in this study (VXA-CoV2-1.1-S) and given as a tablet has begun clinical studies (NCT05067933). In summary, the data presented here demonstrate that oral immunization is a viable strategy to decrease SARS-CoV-2 transmission and disease and should be considered for vaccination efforts that increase global immunity to SARS-CoV-2.

## MATERIALS AND METHODS

### Study design

Male Syrian Hamsters (*Mesocricetus auratus*) approximately 12 to 14 weeks of age with a weight range of 106 to 136 g were sourced from Charles River Laboratory. Animal work was performed at Lovelace Biomedical, with approval from the Institutional Animal Care and Use Committee (IACUC FY20-117E-E5). Hamsters were singly housed in filter-topped cage systems and were supplied with a certified diet, filtered municipal water, and dietary and environmental enrichment. The study was powered to compare viral RNA loads in naïve hamsters exposed to vaccinated, SARS-CoV-2 infected index animals between vaccine groups, where beta was set to 0.2 and alpha=0.05. Assuming an attack rate of 80% infected in the placebo group and a vaccine efficacy of 70%, an N=15 was calculated with continuity correction (*36*). The study was rounded to 16 naïve, 4 index to maintain the 1:4 ratio. Although the study was not powered to directly compare index groups, many statistically significant differences were observed with N=4.

All r-Ad-S vaccinations were given at a dose of 1x10^9^ IU (1:100 of a human dose (*11*)). Oral vaccine was delivered by gavage in 300μl of PBS subsequent to delivery of 300μl 7.5% bicarbonate buffer. IN vaccination was delivered in PBS by pipette, 25μl per nostril, 50μl per animal. The control group received PBS by oral gavage. One group received unadjuvanted recombinant SARS-CoV-2 spike protein, made in insect cells, by 100 μl IM immunization at a dose of 0.1μg per animal (BEI Resources, #NR-52308). All index animals were challenged by IN inoculation of SARS-CoV-2 at approximately 1x10^5^ TCID_50_ per animal in 200 μl (100 μl per nostril) volume seven weeks after the initial vaccination. Index animals were then housed for 24 hours individually before placing in aerosol chamber. Each vaccine-index group had 4 animals, and matched with a corresponding N=16 naïve exposed animals (1:4 ratio of index to naïve, with 1 index animal exposed to 4 naïve in a chamber setup). All animals were euthanized five days post inoculation (index) or aerosol chamber exposure (naïve) for terminal assays.

For serum collection, animals were sedated with a mixture of ketamine (60 to 120mg/kg) and xylazine (5 to 20mg/kg) by intraperitoneal injection to restrain them for all blood draws. Approximately 300 to 500μl blood samples were collected into yellow top serum separator tubes, allowed to clot for 30 min to an hour and processed to serum by centrifugation at 2500 × g for at least 10 min at 2 to 8°C. Serum was aliquoted into a cryovial and stored at -80°C±10°C. BAL was collected by harvesting the right lung, centrifuging at 2000 × g for 5 min, discarding the pellet and freezing at -80°C.

### Vaccine constructs

r-Ad-S is a rAd5 vector containing the gene of the full-length SARS-CoV-2 stabilized S protein, under control of the cytomegalovirus (CMV) promoter. rAd5 vaccine constructs were created based on the published DNA sequence of SARS-CoV-2 publicly available as GenBank Accession No. MN908947.3. The published amino acid sequences of the SARS-CoV-2 S were used to create recombinant plasmids containing transgenes cloned into the E1 region of Adenovirus Type 5 (rAd5) (*37*), using the same vector backbone used in prior clinical trials for oral rAd tablets (*11*, *38*). All vaccines were grown in the Expi293F suspension cell-line (Thermo Fisher Scientific) and purified by CsCl density centrifugation. The spike protein vaccine was provided by BEI Resources, NR-52308, Spike Glycoprotein (Stabilized) from SARS-Related Coronavirus 2, Recombinant from Baculovirus, NR-52308.

### Transmission chamber

The airborne transmission chamber was based on previous transmission work with aerosols^15^ and adapted for hamsters. The chamber consists of multiple subchambers that support unidirectional flow. All chambers were fitted with access doors with air tight seals and appropriate safety features to ensure animals could not interact with fans, sampling, flow, or other features. Approximate dimensions of the first chamber (1) were 4”x10”x9” (length, width, height). The unidirectional flow (5 L/minute) was controlled by regulated house exhaust flow from the third chamber. This drew room air into chamber 1 by a high efficiency particulate air (HEPA) filter (HEPA Vacuum Filter Compatible with Kenmore 86880, EF-2, Panasonic MC-V194H Vacuum Cleaner). Chamber 1 was also fitted with a recirculating fan (ANVISION 40mm × 10mm DC 5V USB Brushless Cooling Fan, Dual Ball Bearing, Model YDM4010B05) to ensure homogeneity of the aerosol prior to transitioning into chambers 2 and 3. The connector chamber (chamber 2) (approximate dimensions of 4”x5”x5”), connected chambers 1 and 3 in order to separate the hamsters, but allows for air passage. Chamber 3 (approximately 10”x10”x9”) housed the naïve hamsters. A wire mesh screen with 0.25”x 0.25” holes was placed at each end of chambers 1 and 3 to prevent hamsters moving to another chamber. Additionally, chamber 2 was raised off the ground to prevent feces or urine from moving between chambers. No bedding was available in any of the three chambers.

### Assessment of infectious SARS-CoV-2 load in lung homogenates

Lung tissue samples from euthanized hamsters infected with SARS-CoV-2 were collected, weighed and homogenized with beads using a Tissue Lyser (Qiagen). Each sample was serially diluted 10-fold in Dulbecco’s Modified Eagle’s Medium containing 2% fetal bovine serum (FBS) and 1% Penicillin-Streptomycin solution. Vero E6 cell monolayers at ≥ 90% confluency in 96-well plates were rinsed with PBS. The plates were inoculated with 100μl of each sample dilution in five technical replicates. Negative control wells contained dilution medium only. The plates were incubated at 37°C and 5% CO_2_ for 72 hours. Cytopathic effect was scored after fixing the cell monolayers with 10% formalin and staining with 0.5% crystal violet. Viral load was determined by TCID_50_/ml of lung homogenate using the Reed and Muench method (*39*). Infectious virus titers in infected lungs were expressed as TCID_50_ per gram of tissue.

### SARS-CoV-2 Surrogate Neutralization Assay

Neutralizing antibodies were measured using a SARS-CoV-2 Surrogate Virus Neutralization Test Kit (GenScript). Hamster serum samples were diluted from 1:20 to 1:500 incubated at a 1:1 ratio with horseradish peroxidase (HRP)-conjugated SARS-CoV-2 RBD protein for 30 min at 37°C. Following incubation, 100 μl was added to human ACE2 pre-coated plates and incubated for 15 min at 37°C. Plates were washed four times with 260μl per well of supplied wash solution, followed by the addition of 100 μl per well of supplied 3,3′, 5,5′ tetramethylbenzidine dihydrochloride (TMB) solution. Plates were developed for 15 min at room temperature in the dark before development was stopped with 50 μl per well of supplied stop solution. Optical densities were measured at 450 nm with a Spectra Max M2 microplate reader.

### IgG ELISA

Purified SARS-CoV-2 S1 protein (GenScript) or Beta and Delta variant S1 proteins (Acro biosciences) in carbonate buffer, pH 9.4, (Thermo Fisher Scientific) were coated onto microtiter plates (Maxisorp, Nunc) at 1 μg/ml and incubated overnight at 4°C before blocking with 100 μl of PBS-0.05% Tween (PBST) + 1% bovine serum albumin (BSA) for 1 hour. Serum samples were serially diluted in PBST. After a 2 hour incubation at room temperature the plates were washed 3x with PBST, followed by the addition of 100 μl per well of 1:3000 goat anti-hamster IgG-HRP (Thermo Fisher Scientific) in PBST + 1% BSA. Plates were incubated at room temperature for 1 hour before washing three times with PBST. Next, TMB substrate (Rockland) was added at a concentration of 50μl per well. The plates were developed for 10 min, then stopped with 50μl per well of 2M sulfuric acid. Optical densities were measured at 450nm with a Spectra Max M2 microplate reader.

### IgA MSD Assay

S1 protein was biotinylated according to manufacturer’s instructions (EZ-link, Thermo Fisher Scientific), and was conjugated to a U-Plex MSD linker (Mesoscale Discovery). The linked S1 protein was coated on 2-spot U-plex 96 well plates (Mesoscale Discovery) for a final concentration of 66nM per well and incubated overnight. The next day, the plates were blocked with PBS-T for 1 hour prior to the addition of hamster serum or BAL fluid. Samples from each individual hamster acquired on days 0, 28, and 55 were quantified on the same plate. The serum samples were diluted to 1:200 and the BAL samples were added to the plate neat. Following a 2 hour sample incubation, the plate was washed and SULFO-TAG (MSD) anti-hamster IgA (Brookwood Biomedical) detection antibody was added. The plate was washed and 1X MSD read buffer was added and each plate was analyzed using MSD QuickPlex.

### SARS-CoV-2 Challenge

All animals were challenged by IN inoculation of 1x10^5^ TCID_50_/animal SARS-CoV-2 (isolate USA-WA1/2020) at 100μl per nostril (for a total volume of 200μl) eight weeks post initial vaccination. SARS-CoV-2, isolate USA-WA1/2020, was sourced from BEI Resources. The complete genome has been previously sequenced for the original isolate: GenBank accession number MN985325, after one passage in Vero E6 cells: GenBank accession number MT020880, and after four passages in Vero E6 cells: GenBank accession number MT246667. It was propagated in Vero E6 African Green Monkey kidney cells (BEI Resources, catalog #N596) at the University of Texas Medical Branch (UTMB) and virus was stored in a biosafety level 3 compliant facility.

### Collection of nasal swabs

Plasdent Dentistry Maxapplicators with 0.5mm ultrafine non-linting, non-absorbable fiber head tips were used for nasal swab collections. Swabs were inserted approximately 2 to 4mm into the naris of an anesthetized hamster, removed and cut at the hinge point. Swabs were then placed into Safe-Lock Eppendorf tubes and flash frozen at -80° ±10°C until RNA isolation.

### Detection of SARS-CoV-2 genomic RNA in nasal swabs and lung homogenates by qRT-PCR

Lung samples were weighed and homogenized with beads using a Tissue Lyser (Qiagen) in 1 ml of TRI reagent, before RNA was isolated and purified from tissue samples using the Direct-Zol 96- RNA kit (Zymo Research). Copies of the SARS-CoV-2 N gene were measured by qRT-PCR TaqMan Fast Virus 1-step assay (Applied Biosystems). SARS-CoV-2 specific primers and probes from the 2019-nCoV RUO Assay kit (Integrated DNA Technologies) were used: (L Primer: TTACAAACATTGGCCGCAAA; R primer: GCGCGACATTCCGAAGAA; probe:6FAM-ACAATTTGCCCCCAGCGCTTCAG-BHQ-1). Reactions were carried out on a Stratagene MX3005P or BioRad CFX384 Touch instrument according to the manufacturer’s specifications. A semi-logarithmic standard curve of synthesized SARS-CoV-2 N gene RNA (LBRI) was obtained by plotting the Ct values against the logarithm of cDNA concentration and used to calculate SARS-CoV-2 N gene in copies per gram of tissue.

### Gross pathology scoring

Gross necropsy observations of the lung were recorded in Provantis using consistent descriptive terminology to document location(s), size, shape, color, consistency, and number. Gross observations included a severity grade for red discoloration of the lung (likely to be associated with pneumonia) based on a 0 to 4 scale indicating percent of whole lung affected: none (no grade), minimal (1), mild (2), moderate (3), marked (4) correlating to 0, 1 to 25, 26 to 50, 51 to 75, and 76 to 100% affected, respectively.

### Clinical protocol

A phase 1 clinical study (https://clinicaltrials.gov/show/NCT04563702) was designed to evaluate the safety and immunogenicity of an oral spike and nucleocapsid (N) protein vaccine (termed VXA-CoV2-1) in 35 individuals at two different doses (1 × 10^10^ IU and 5 × 10^10^). Five sentinel volunteers were dosed first and after a week of monitoring for vaccine-induced toxicities, the remaining volunteers in the treated cohort were randomized with 4 placebo controls. Only 5 individuals in the low dose group were boosted; all other participants were given 1 dose of VXA-CoV2-1. In terms of demographics, 66% of participants were male, 57% were white, 20% were African-American, 14% were Hawaiian or Pacific Islander, and 9% were other. 29% of participants reported being Hispanic or Latino. Participants were between the ages of 18 and 53.

### Study approval

The study was conducted in accordance with applicable Good Clinical Practice guidelines, the United States Code of Federal Regulations, and the International Conference on Harmonization guidelines. Institutional Review Board (IRB) approval was obtained from the Aspire IRB (Protocol #20202876) before study-specific screening and enrollment of participants. Informed consent was obtained from all participants after discussion of the study procedures and potential risks.

### Measurement of vaccine-induced IgA antibodies mucosal samples

Nasal samples were collected according to the package insert for the Nasosorption FX-I /SAM devices (Mucosal Diagnostics) and extracted by centrifugation in a 1X PBS with 0.02% azide solution. Saliva samples were collected by centrifugation after having each participant chew on a cotton swab for 45 s and transferred to a salivette (Sarstedt). IgA was measured with a SARS-CoV-2 specific (panel 2) and Coronavirus panel 3 MSD immunoassay. Plates were blocked, washed, and incubated with sample and detection antibody according to the manufacturer’s instructions. Samples were diluted at 1:10 and 1:100 in Diluent 100. Plates were read on a Meso QuickPlex instrument. Sample antibody concentrations were reported in arbitrary units (AU) per ml as calculated from a standard curve supplied with the kit.

Due to the variability in sampling the human mucosa, samples were normalized using an ELISA for the detection of total IgA. Briefly, purified anti-human IgA monoclonal antibody (mAb) MT57 (Mabtech) was coated onto 96-well Maxisorp plates (Thermo Fisher Scientific) at 2μg/ml in PBS and incubated overnight at 4°C. Plates were washed with 1X PBS + 0.1% Tween-20 (PBST) and blocked with PBST + 1% BSA for 1 hour at room temperature. Following a wash step, saliva and eluted nasal samples were diluted 1:100 in PBST and serially diluted 1:3 down the plate. Human IgA (Sigma-Aldrich) was used to create a standard curve starting at 200ng/ml and serially diluted two-fold 7 times. The samples and standards were transferred to the coated plate and incubated for 2 hours at room temperature. After a wash step, a 1:1000 dilution of anti-human IgA mAb MT20 ALP conjugate (Mabtech) was added to the nasal samples. The plates were incubated at room temperature for 1 hour followed by washing. Plates with nasal samples developed for 1 hour in the dark with para-Nitrophenylphosphate (pNPP) substrate (Mabtech) and ODs were measured at 405nm with a Spectra Max M2 microplate reader. Plates with saliva samples were developed in the dark with an HRP substrate (Rockland) and were stopped with sulfuric acid (Honeywell). ODs were measured at 450nm. The concentration of total IgA in human mucosal samples was generated by a standard curve. A normalization factor was applied by dividing the post vaccination sample by the pre-vaccination sample. Data analysis was performed in GraphPad Prism Software.

### Statistical Analysis

All raw, individual-level data are presented in data file S1. The methods used for determining significance were a one- or two-way ANOVA and Dunnett’s multiple comparisons test or Fisher’s exact test. For Fig. 1C, a one-way ANOVA and Tukey’s multiple comparisons was used for intra-variant comparisons. Kruskal-Wallis test and Dunn’s multiple comparisons was done for Fig. 2D, where the majority of data was below the limit of detection.

## Supplementary Materials

### Other Supplementary Material for this manuscript includes the following:

MDAR Reproducibility Checklist
