# Supplementary material for: Adenovirus type 5 SARS-CoV-2 vaccines delivered orally or intranasally reduced disease severity and transmission in a hamster model
Source: Sci Transl Med. 2022 May 5:eabn6868. doi: 10.1126/scitranslmed.abn6868 (PMC9097881; doi:10.1126/scitranslmed.abn6868)
Supplement: Supplementary file 1 — Figs. S1 to S3 Table S1 [file scitranslmed.abn6868_sm.pdf]

Science Translational Medicine
AAAS

Supplementary Materials for

Adenovirus type 5 SARS-CoV-2 vaccines delivered orally or intranasally reduced disease severity and transmission in a hamster model

Stephanie N. Langel *et al.*

Corresponding authors: Stephanie N. Langel, stephanie.langel@duke.edu;
Sean N Tucker, stucker@vaxart.com

DOI: 10.1126/scitranslmed.abn6868

The PDF file includes:

Figs. S1 to S3
Table S1

Other Supplementary Material for this manuscript includes the following:

MDAR Reproducibility Checklist
Data file S1

Supplementary Materials for

Adenovirus type 5 SARS-CoV-2 vaccines delivered orally or intranasally reduced disease severity and transmission in a hamster model

Stephanie N. Langel[1*], Susan Johnson[2*], Clarissa I. Martinez[2], Sarah N. Tedjakusuma[2], Nadine Peinovich[2], Emery G. Dora[2], Philip J. Kuehl[3], Hammad Irshad[3], Edward G. Barrett[3], Adam Werts[3] and Sean N Tucker[2±]

Correspondence to: stephanie.langel@duke.edu (S.N.L.); stucker@vaxart.com (S.N.T.)

This PDF file includes:

Figs. S1 to S3
Tables S1

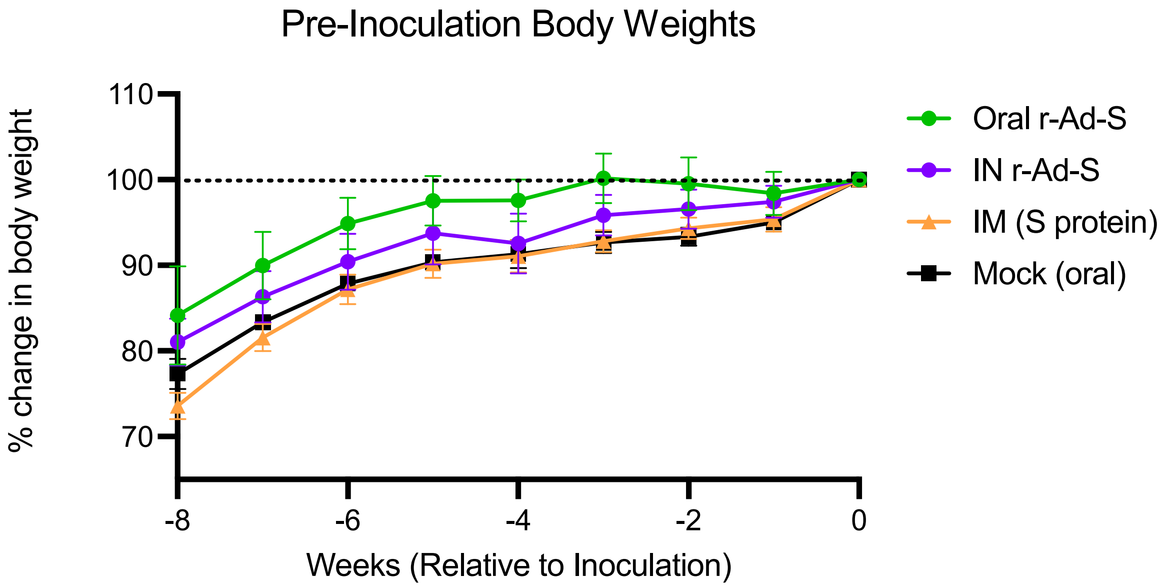

Fig S1. All animals gained weight prior to severe acute respiratory syndrome coronavirus 2 (SARS-CoV-2) inoculation. Animals (n=4 per group) were weighed weekly and data were graphed as a percent of day 0 (SARS-CoV-2 inoculation day) in hamsters that were vaccinated with oral r-Ad-S, intranasal (IN) r-Ad-S, intramuscular (IM) spike (S) or mock (oral) vaccines. Error bars represent the standard error of the mean (SEM). Ad, adenovirus.

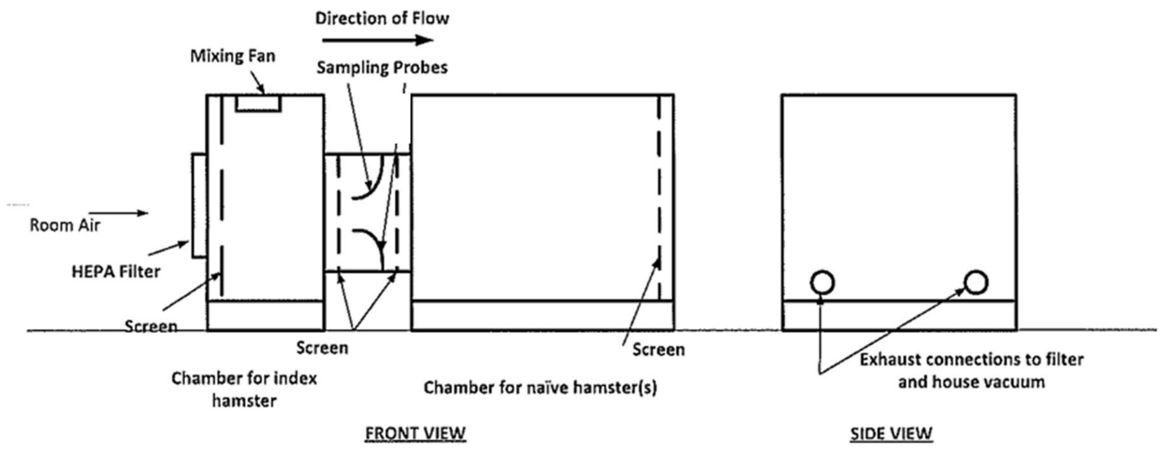

Fig S2. Airflow transmission chamber. HEPA, high efficiency particulate air. The chambers included a chamber for the index hamster (left), a connector chamber (middle), and a chamber for the naïve hamster(s) (right).

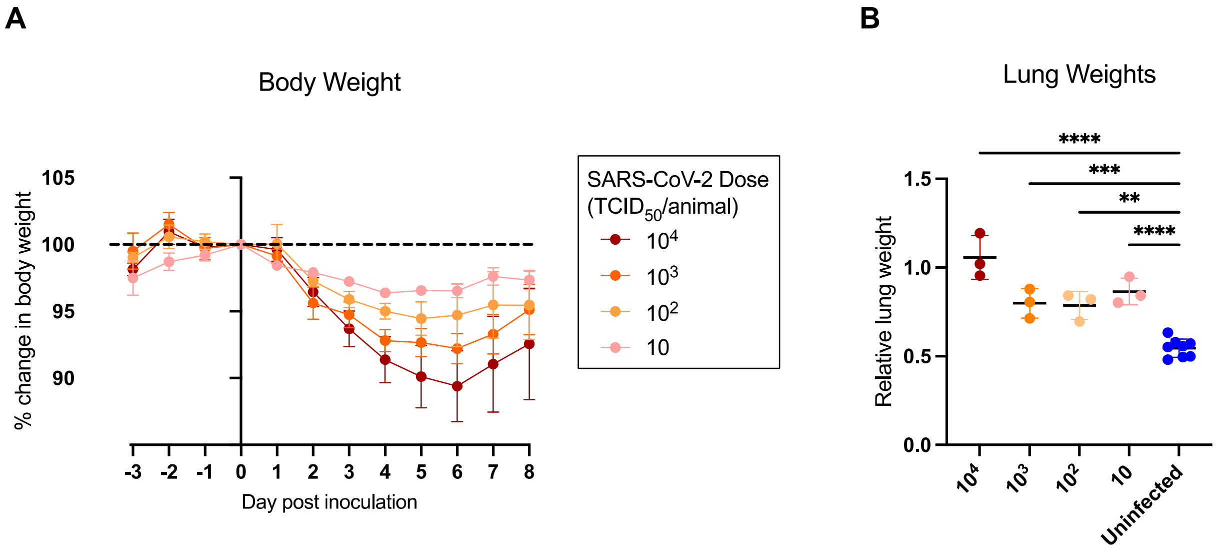

Fig S3. Disease severity varied directly in accordance with the viral titer at inoculation. **(A)** Hamsters (n=3 per group) were inoculated with 10, 10^2, 10^3, or 10^4 Median Tissue Culture Infectious Dose ($TCID_{50}$) of SARS-CoV-2 and body weights were measured for 8 days. The red arrow signifies the day of SARS-CoV-2 inoculation. **(B)** Lung weight was measured as a percent of body weight at day 8 post inoculation (n=8 hamsters were used as uninfected controls). Error bars represent the SEM. $^{**}P<0.01$, $^{***}P<0.001$, $^{****}P<0.0001$.

Table S1. Outcome table for animals at or above 1×10^5 gene copies of SARS-CoV-2 nucleocapsid protein. Significance was determined by Fisher's exact test. NA, not applicable.

**Day 1. Gene Copies 1×10^5 or Above**						
**Group**	**Positive**	**Negative**	**p versus oral**	**Significant**	**p versus mock**	**Significant**
Oral rAd-S	3	13	NA	NA	0.011	Yes
Intranasal rAd-S	0	16	0.22	No	0.0001	Yes
Spike protein vaccine	10	6	0.029	Yes	1	No
Mock	11	5	0.011	Yes	NA	NA
**Day 3. Gene Copies 1×10^5 or Above**						
**Group**	**Positive**	**Negative**	**p versus oral**	**Significant**	**p versus mock**	**Significant**
Oral rAd-S	11	5	NA	NA	0.043	Yes
Intranasal rAd-S	7	9	0.2852	No	0.0008	Yes
Spike protein vaccine	16	0	0.043	Yes	1	No
Mock	16	0	0.043	Yes	NA	NA
